# Baseline glucocorticoids alone do not predict reproductive success across years, but in interaction with enzymatic antioxidants

**DOI:** 10.1002/ece3.11193

**Published:** 2024-04-01

**Authors:** Lucia Mentesana, Stefania Casagrande, Michaela Hau

**Affiliations:** ^1^ Max Planck Institute for Biological Intelligence Seewiesen Germany; ^2^ University of Konstanz Konstanz Germany

**Keywords:** body condition, corticosterone, fitness, glucocorticoids, oxidative stress, physiological network

## Abstract

Glucocorticoids are known to adjust organismal functions, such as metabolism, in response to environmental conditions. Therefore, these hormones are thought to play a key role in regulating the metabolically demanding aspects of reproduction, especially in variable environments. However, support for the hypothesis that variation in glucocorticoid concentrations predicts reproductive success is decidedly mixed. Two explanations may account for this discrepancy: (i) Glucocorticoids might not act independently but could interact with other physiological traits, jointly influencing reproduction, and (ii) such an association could become apparent primarily in challenging environments when glucocorticoid concentrations increase. To address these two possibilities, we determined natural variation in circulating baseline glucocorticoid concentrations in parental great tits (*Parus major*) alongside two physiological systems known to be related with an individual's metabolism: oxidative status parameters (i.e., concentrations of pro‐oxidants, dietary, and enzymatic antioxidants) and body condition. These systems interact with glucocorticoids and can also influence reproductive success. We measured these variables in two breeding seasons that differed in environmental conditions. When accounting for the interaction of baseline glucocorticoids with other physiological traits, we found a positive relationship between baseline glucocorticoids and the number of fledglings in adult great tits. The strength of this relationship was more pronounced for those individuals who also had high concentrations of the enzymatic antioxidant glutathione peroxidase. When studied independently, glucocorticoids were not related to fitness proxies, even in the year with more challenging environmental conditions. Together, our study lend to support the hypothesis that glucocorticoids do not influence fitness alone, but in association with other physiological systems.

## Graphical abstract

Glucocorticoids are thought to play a key role in regulating the metabolically demanding aspects of reproduction, especially in variable environments. However, evidence supporting this idea is mixed. Our study shows that glucocorticoids do not influence fitness independently, but in association with other physiological systems.

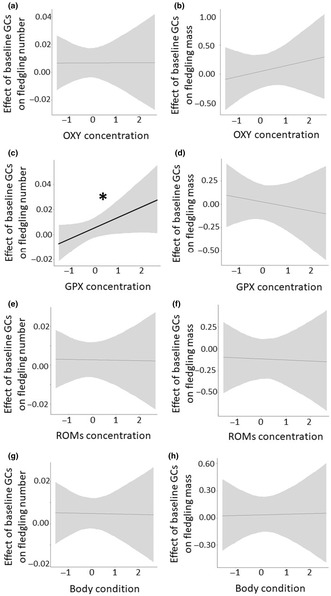

## 1 INTRODUCTION

Organisms need to continuously adjust and respond to fluctuating environmental conditions. Physiological signals play a crucial role in enabling organisms to cope with such changes. These signals integrate information from the environment, the genome, and an individual's internal state to mediate a phenotypic response (Ricklefs & Wikelski, 2002; Zera & Harshman, 2001). However, the specific physiological traits that contribute to effective coping mechanisms, thus promoting an individual's fitness, are still an open question in evolutionary biology.

Maximizing reproductive success, especially in challenging environmental conditions, demands a substantial energetic investment. Consequently, one might expect that physiological traits related to energy metabolism play a key role in linking these traits to an individual's reproductive fitness. Glucocorticoid hormones have a critical function in adjusting organismal metabolism and functioning in response to environmental conditions (as reviewed by Hau et al., 2016; Romero & Wingfield, 2016). At low baseline concentrations, glucocorticoids mediate responses to predictable daily metabolic demands and life‐history stages by mobilizing energy reserves and influencing behavioral performance (Landys et al., 2006; McEwen & Wingfield, 2003; Romero et al., 2009; Sapolsky et al., 2000). Due to the phenotypic processes supported by baseline glucocorticoids, numerous studies have attempted to relate it to fitness parameters. Despite the efforts to establish a clear link between glucocorticoids and fitness, a consensus result of the relationship between baseline glucocorticoids and fitness has not yet emerged in vertebrate species (Beehner & Bergman, 2017; Bonier, Martin, et al., 2009; Bonier, Moore, et al., 2009; Breuner et al., 2008; Crespi et al., 2013; Schoenle et al., 2021).

The lack of consensus may stem from two issues. First, some studies solely focus on this hormone, but recent work suggests that phenotypic responses result from the interaction of multiple physiological systems (Cohen et al., 2012; Fuxjager et al., 2023; Martin et al., 2011; Ricklefs & Wikelski, 2002). Glucocorticoids (also referred as “integrators” in “physiological regulatory networks” by Cohen et al., 2012) have widespread effects and interact with other physiological systems, such as oxidative status and body condition (i.e., body mass relative to body size) among others. They can negatively impact an individual's oxidative status by increasing the production of free radicals and/or by reducing the concentration of antioxidants, which protect tissues from damage, particularly when glucocorticoids are present in high concentrations (Costantini, Marasco, et al., 2011; Haussmann & Marchetto, 2010). Additionally, glucocorticoids are often higher in individuals with lower body condition (reviewed by e.g., Landys et al., 2006). The interaction between glucocorticoids, oxidative status markers, and body condition can be associated with an individual's fitness (e.g., Casagrande & Hau, 2018; Jenni‐Eiermann et al., 2008; Lendvai et al., 2014; Vágási et al., 2020; Vágási, Pătraș, et al., 2018). Therefore, one could hypothesize that glucocorticoids form an interacting physiological system together with oxidative status and body condition, and that the interactions between glucocorticoids and these two components determine reproductive fitness performance. To test if glucocorticoids function together with oxidative status markers and body condition, researchers have used different approaches: Four studies tested the relationships among glucocorticoids, oxidative status markers and body condition parameters in unmanipulated individuals from natural bird populations. A positive relationship between baseline glucocorticoids and oxidative (lipid) damage was reported in house sparrows (*Passer domesticus*) with high body condition (Vágási et al., 2020), while a negative relationship between baseline glucocorticoids and oxidative stress (defined by the authors as high antioxidant capacity and low oxidative damage) was found in female, but not in male, tree swallows (*Tachycineta bicolor*, Ouyang et al., 2016). In contrast, no correlation between glucocorticoids and oxidative status markers was found in female European starlings (*Sturnus vulgaris*; Fowler et al., 2018) or in both sexes of cooperatively breeding superb starlings (*Lamprotornis superbus*, Guindre‐Parker & Rubenstein, 2018). Of these four studies, three tested the hypothesis that glucocorticoids influence reproductive fitness traits while simultaneously accounting for the action of oxidative status markers and body condition (i.e., by accounting for the overall physiological condition of individuals; Fowler et al., 2018; Guindre‐Parker & Rubenstein, 2018; Ouyang et al., 2016) and found no support. To date, only one study tested if the interaction of glucocorticoids with the other two physiological systems jointly influenced fitness (Fowler et al., 2018) and reported no association. Hence, in free‐living bird populations, there is scarce evidence for glucocorticoids acting jointly with oxidative status and body condition to maximize reproductive fitness.

A second possible explanation for the lack of consensus across studies is that the relationship between glucocorticoids and reproductive success may vary depending on the environmental context (Bonier & Martin, 2016; Dantzer et al., 2016). Specifically, baseline glucocorticoids are expected to increase during challenging times to support reproductive performance (“Cort‐adaptation hypothesis”; Bonier, Moore, et al., 2009). Two recent multiyear studies conducted in female birds supported this hypothesis by finding a positive relationship between baseline glucocorticoids and reproductive fitness, particularly during years with adverse weather conditions (*Cyanistes caeruleus*; Henderson et al., 2017; *T. bicolor*; Vitousek, Taff, Hallinger, et al., 2018). However, no such relationship was observed in female European starlings (Fowler et al., 2018), and a negative relationship was found in great tits (*Parus major*) of both sexes when tested during the breeding season across years with varying environmental conditions (Ouyang et al., 2013). The relationship between baseline glucocorticoids and fitness under variable environmental conditions therefore remains a topic of debate, likely owing to the complexity of environments inhabited by free‐living organisms. Improving our understanding requires assessing such relationships across various environmental contexts.

In this study, we examined the relationship between baseline glucocorticoids and reproductive fitness within the framework of the two hypotheses proposed to explain the lack of consensus. (i) glucocorticoids act jointly with oxidative status markers and body condition, interacting to predict reproductive success, and (ii) glucocorticoids predict reproductive success depending on the environmental context. For the first hypothesis, we predict that baseline glucocorticoids will be positively related to pro‐oxidants and negatively related to antioxidants and body condition. In this context, we anticipate that individuals with low baseline glucocorticoids, but high concentrations of antioxidants and favorable body condition will experience higher reproductive success. Concerning the second hypothesis, we predict that baseline glucocorticoids will be associated with reproductive success in years with less favorable environmental conditions. For this, we measured circulating baseline glucocorticoid concentrations, oxidative status markers (including concentrations of pro‐oxidants, non‐enzymatic, and enzymatic antioxidants), and body condition (body mass relative to tarsus length) in free‐living great tits during two breeding seasons with different environmental conditions (i.e., one favorable and one unfavorable). Great tits are excellent model species for studying evolutionary questions because their reproductive success exhibits marked variation among individuals across years due to individual and environmental characteristics (Perrins & Moss, 1975). Moreover, they reproduce in nest boxes placed in the field, allowing us to quantify reproductive success in natural settings. We tested the hypothesis that glucocorticoids form an interacting physiological system with oxidative status markers and body condition, jointly influencing reproductive success. To do this, we (i) investigated the relationships between the five physiological markers, (ii) explored whether glucocorticoids predict reproductive success while accounting for the influence of oxidative status markers and body condition, and (iii) tested whether glucocorticoids interact with oxidative status markers and body condition to jointly predict reproductive success. We then examined the possibility that the influence of glucocorticoids on reproductive success is dependent on environmental conditions by comparing two breeding seasons that differed in environmental conditions.

## 2 MATERIALS AND METHODS

### 2.1 Study site, standard protocols, and reproductive success

We conducted this study in a nest box population in the Dellinger Buchet, which is a mosaic of deciduous and coniferous forest patches in Southern Germany (Bavaria; 48°03′ N, 11°13′ E, 620 m above sea level) from April to July in 2015 and 2016. We monitored nest boxes so that we could determine when the first egg was laid and the first chick hatched (hatching day = nestling day 0; see Mentesana et al., 2019, 2021 for a detailed description of the study site and standard protocol for monitoring nest boxes). For this study, we only considered first clutches since only few great tits produce second clutches in a year.

On nestling day 9, we captured parents inside their nest boxes between 08:00 and 12:00 am. For this, we used a remote‐controlled trap that closed the nest entrance after parents entered to feed their young. Within 3 min of capture (mean ± SD: 2.36 ± 0.51 min), we collected a blood sample (max. 80 μL) to determine each parent's baseline corticosterone concentrations and oxidative status. We then identified each parent individually with a numbered aluminum ring and three colored plastic split rings. We recorded body mass (to the nearest 0.1 g), tarsus and wing length (both traits to the nearest 0.1 mm). Of these three parameters measured, we used the two traits with the highest correlation to estimate body condition (Peig & Green, 2009): body mass and tarsus length (*r* = .41; *p*‐value < .00). This “scaled mass index” represents a measure of body mass relative to body size, and can serve as a proxy for the amount of energy reserves an individual carries (Peig & Green, 2009). Since this population was established in 2015, we could not collect sufficient information regarding the age of the birds. Consequently, birds of various ages and breeding experiences were analyzed together. We then released the adults at the site of capture.

We recorded two proxies of reproductive success: fledgling number and fledging mass. We determined fledgling number by monitoring nests every 5 days until fledging date (~day 20 after hatching). On day 15 after hatching, we measured fledgling body mass as a key predictor of post‐fledging survival in great tits (e.g., Tinbergen & Boerlijst, 1990).

### 2.2 Assessment of environmental conditions

Great tit parents rely mainly on caterpillars to feed their young (Gibb, 1950). Weather conditions can influence caterpillar development and growth, thus influencing the amount of food available during the breeding season (Perrins, 1991; Van Noordwijk et al., 1995). We therefore obtained information of rainfall (mm) every 5 min from a meteorological station less than 5 km from our field site (Oberpfaffenhofen; 48°05′ N, 11°16′ E, 583 m above sea level) and recorded environmental temperature (accuracy of ±0.5°C) every hour from 12 i‐buttons (DS9093A+ Thermochron iButton) placed in different locations in the forest. We then estimated mean rainfall and mean temperature experienced by each brood from day 1 to 20 after hatching.

### 2.3 Corticosterone hormone analysis

We determined plasma corticosterone concentrations using enzyme immunoassay kits (Lot No: 2015: 12041402C, 2016: 12021512B; Enzo Life Sciences GmbH, Germany) following a double diethyl ether extraction of 10 μL plasma aliquots for baseline levels (see Baugh et al., 2014 for further details on this protocol). We then re‐dissolved each sample, a blank and two positive controls (i.e., each with 14 μL of stripped‐chicken plasma at a concentration of 20 ng/mL) in 280 μL of assay buffer. After overnight reconstitution, we took all samples through the entire assay. We added duplicates of 100 μL to individual wells. We randomized the position of the samples across 21 plates. The intra‐assay coefficients of variation, determined from two positive controls per assay, was (mean ± SD) = 10.57 ± 7.95%, and the inter‐assay variation, determined from the first positive control of each assay, was (mean ± SD) = 11.55 ± 8.86%.

### 2.4 Oxidative status analyses

We measured the concentrations of reactive oxygen species (ROMs; expressed as mM H_2_O_2_ equivalents) in the plasma of adults by using the d‐ROMs test kit (Diacron International SRL, Grosseto, Italy), following Costantini et al. (2006). We measured plasma concentrations of non‐enzymatic antioxidants (OXY; expressed as mM HOCl neutralized) using the OXY‐Adsorbent test (Diacron International SRL, Grosseto, Italy), following Costantini et al. (2006). We determined the activity of the enzymatic antioxidant glutathione peroxidase (GPX, expressed as U/mL) present in red blood cells using the Ransel assay (Randox Laboratories, Germany), following Costantini, Monaghan, et al. (2011).

### 2.5 Statistical analysis

To test the hypothesis that glucocorticoids form an interacting physiological system with oxidative status markers and body condition, jointly influencing reproductive success, we performed three stepwise and complementary statistical tests. For this, we pooled the data from the 2 years because reproductive success did not differ between years. First, to investigate if there is phenotypic integration between the five physiological variables, we explored the relationships between glucocorticoids, the three parameters of oxidative status, and body condition by calculating pairwise Pearson correlation coefficients. This correlation was calculated by pooling the sexes and the years, given that we found similar results when analyzing each sex and year separately. Second, we explored whether glucocorticoids predict reproductive success while accounting for the influence of oxidative status markers and body condition by running two linear mixed effect models. In each model, we fitted fledgling number and mass corrected by clutch size (i.e., using the residuals of a linear regression of fledgling number or mass and brood size on day 15 as the dependent variable) as response variables, respectively, and included all five physiological variables as covariates. Third, to test if glucocorticoids interact with oxidative status markers and body condition to jointly predict reproductive success, we ran four linear mixed models. In two of the models, we fitted fledgling number and mass (both corrected for clutch size) as response variables in separate models and included the interactions between baseline corticosterone and each of the three parameters of oxidative status (i.e., OXY, GPX and ROMs) as covariates. In the other two models, we used the same response variables but included the interaction between baseline corticosterone and body condition as a covariate. Note that, in order to examine the specific predictions expected from each component of the oxidative system regarding their interaction with glucocorticoids and their correlation with reproductive fitness, or to interpret the results from a biological standpoint, we had to test the interaction between baseline glucocorticoids and each physiological system separately (i.e., oxidative status markers and body condition) to prevent a single overfitted model. In all four models, we added “nest ID” as a random factor to account for unmeasured factors that might have influenced fledglings growing up in the same nest box, such as genetic, maternal or microenvironmental factors. We initially also included “bird ID” as a random factor to control for multiple tests done on the sample individual, but since it did not explain any variance, we excluded it from all final models.

We next tested the hypothesis that baseline corticosterone influences reproductive fitness traits depending on environmental conditions. To do this, we ran two linear mixed‐effect models, with fledgling number and mass (both corrected for clutch size) as response variables. We included baseline corticosterone as a covariate and “year” as a fixed factor, along with their interaction. Additionally, we added “nest ID” as a random factor to account for effects of the nest environment effects.

For all statistical analyses, we used R version 4.2.1 (R Core Team, 2013). We performed the correlation test using the R‐package “psych.” We ran generalized and linear mixed effect models using the “lme4” and “arm” packages in a Bayesian framework with flat priors. We mean‐centered all continuous explanatory variables to facilitate comparisons across traits. We assumed a Gaussian error distribution, which was confirmed for all response variables after visual inspection of model residuals (Korner‐Nievergelt et al., 2015). We subsequently used the *sim* function to simulate values from the posterior distributions of model parameters. Based on 10,000 simulations, we extracted the 95% Bayesian credible interval (CrI) around the mean. We assessed statistical support based on the estimated effect sizes (i.e., mean estimates), credible intervals, and posterior probabilities for each parameter (Gelman et al., 2012; Gelman & Hill, 2007).

## 3 RESULTS

We sampled 109 adults over two breeding seasons (*N* females: 2015 = 8, 2016 = 42; *N* males: 2015 = 14, 2016 = 37; total *N* pairs: *N* = 60), of which eight were captured twice. We obtained proxies for reproductive success (i.e., fledgling number and mass) for 185 chicks (2015 = 41, 2016 = 144) from 40 nests (2015 = 11, 2016 = 31).

The nestling phase of 2015 was on average less rainy (*F*‐test_(df:58)_: 8.82, *p*‐value = .01; mean ± SE 2015: 3.66 ± 0.34; 2016: 4.93 ± 0.20 mm) and appeared somewhat warmer (although this difference was not statistically significant: *F*‐test_(df:58)_: 0.59, *p*‐value = .44; mean ± SE 2015: 12.21 ± 0.37; 2016: 11.86 ± 0.21°C) compared to 2016. Despite environmental conditions being different, fledgling number and mass did not differ between years (Table S1). Baseline corticosterone, ROM concentrations and body condition of the birds did not differ between years, but parents had a higher concentration of OXY and lower concentrations of GPX in the year with less rain compared with the year with more rain (Table 1).

**TABLE 1:** Results from linear models testing the differences in each of the five physiological parameters measured in adult great tits in the 2 years (2015/less rainy vs. 2016/rainier).

	Baseline corticosterone	OXY	GPX	ROMs	Body condition
Fixed factors *β* (95% CrI)					
Intercept	5.17 (4.02; 6.31)	0.51 (0.09; 0.92)	−0.48 (−0.89; −0.07)	−0.08 (−0.51; 0.35)	0.30 (−0.84; 0.08)
Year	−0.19 (−1.44; 1.11)	**−0.64 (−1.10; −0.17)**	**0.60 (0.14; 1.05)**	0.10 (−0.38; 0.57)	−0.38 (−0.84; 0.08)

*Note*: In 2016, parents had lower concentration of nonenzymatic antioxidants in plasma (OXY) and higher concentration of enzymatic antioxidants in red blood cells (GPX). Other oxidative status markers: ROMs, reactive oxygen metabolites in plasma.

Bold values are posterior probabilities > 99%.

### 3.1 Do glucocorticoids form an interacting physiological system with oxidative status markers and body condition, and jointly predict reproductive success?

**FIGURE 1:**
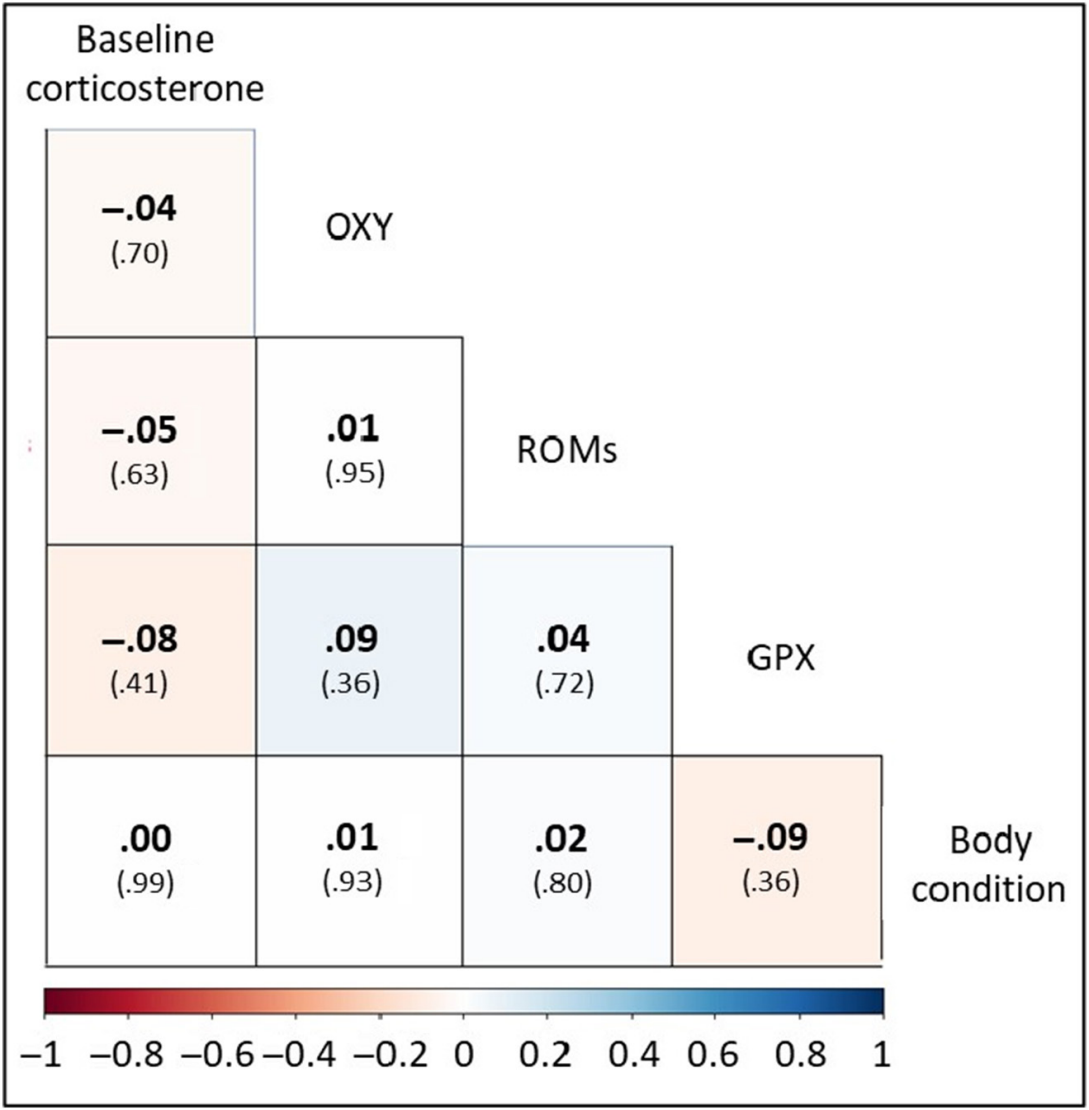
Pearson correlation coefficients and *p*‐values within brackets for all physiological traits measured in great tits during the breeding seasons of 2015 and 2016. We found no statistical evidence for relationships between the five physiological variables. Oxidative status markers: GPX, enzymatic antioxidant in red blood cells; OXY, nonenzymatic antioxidants in plasma; ROMs, reactive oxygen metabolites in plasma.

We found no statistical evidence for bivariate associations between baseline corticosterone, oxidative status markers, and body condition in adult great tits sampled during the breeding seasons of 2015 and 2016 (Figure 1).

**FIGURE 2:**
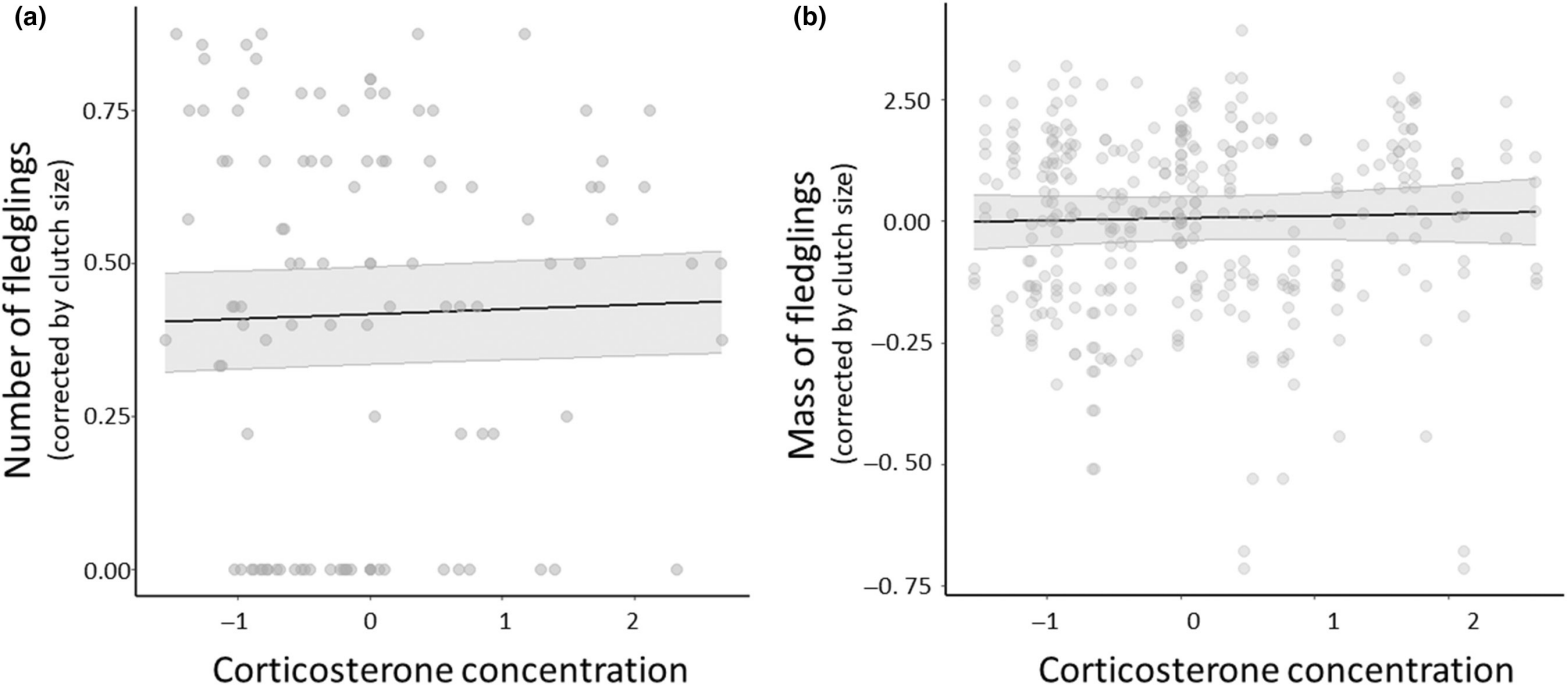
We detected no relationships were between the concentration of baseline corticosterone (ng/mL) and (a) the number or (b) the mass of fledglings (g) after statistically accounting for the effect of oxidative status markers and body condition. Black lines represent mean estimates, gray shaded areas show the 95% credible intervals, and gray circles show the individual raw data.

Although we found no statistical evidence that baseline glucocorticoids were correlated with oxidative status parameters and body condition, these physiological traits could still have jointly influenced an individual's reproductive performance. However, our findings indicate that baseline glucocorticoid concentration did not predict the number (mean = 0.01 [95% credible interval = −0.00; 0.02]; posterior probability >95%; Figure 2a) or mass (mean = 0.05 [95% credible interval = −0.16; 0.25]; posterior probability >67%; Figure 2b) of fledglings when statistically controlling for the effect of oxidative status markers and body condition (Table S2). We also found no statistical evidence for a relationship between oxidative status markers and body condition with reproductive success (Table S2).

**FIGURE 3:**
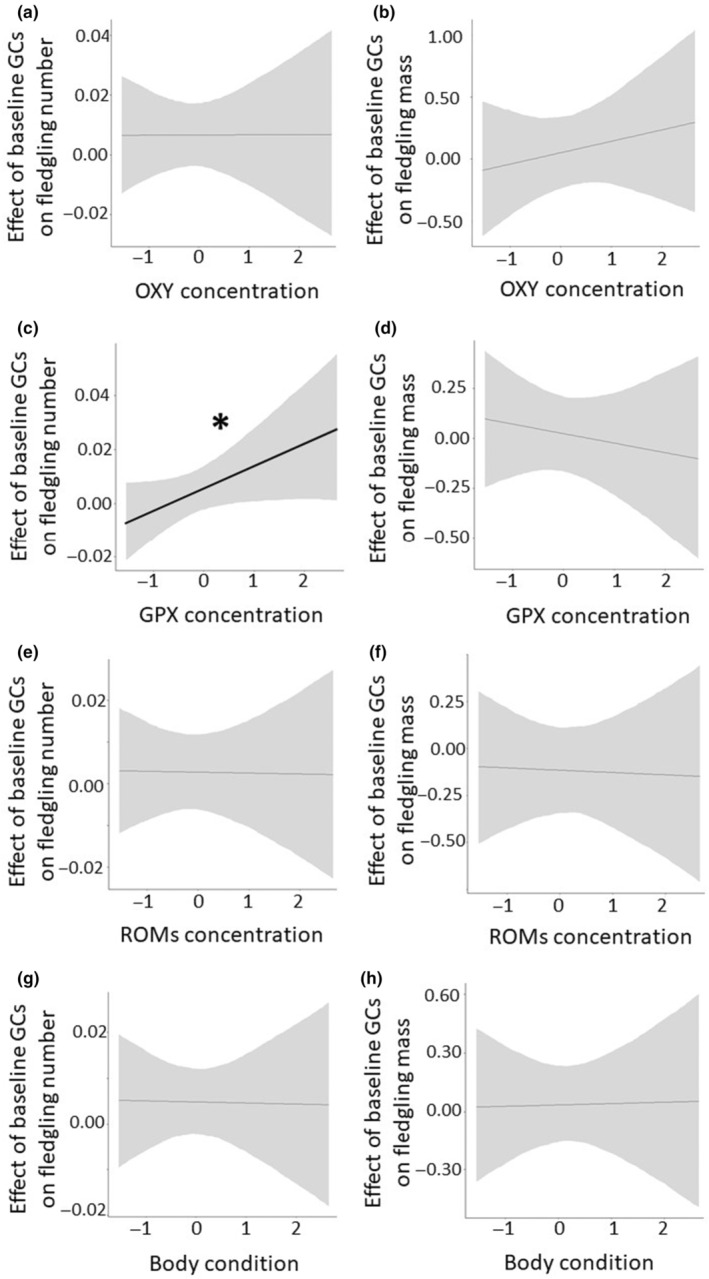
Influence of oxidative status markers and body condition on the relationship between baseline glucocorticoids (GCs) and reproductive success (number and mass of the fledglings, both corrected by clutch size). When adult great tits had high concentrations of GPX, baseline corticosterone was positively related to the number of fledglings (indicated with a thick black line and an asterisk). For the rest of the parameters, we found no statistical evidence for an interaction. Black lines represent mean estimates, and gray shaded areas show the 95% confidence intervals. We mean‐centered all physiological traits to facilitate comparisons across them. Oxidative status markers: GPX, enzymatic antioxidant in red blood cells; OXY, nonenzymatic antioxidants in plasma; ROMs, reactive oxygen metabolites in plasma.

When accounting for the interaction between baseline corticosterone and oxidative status parameters, the former was positively associated with the number of fledglings (mean = 0.01 [95% credible interval = 0.00; 0.02]; posterior probability >99%; Table S3). Additionally, we observed an interaction between baseline glucocorticoids and GPX: when adult great tits had high concentrations of GPX, baseline glucocorticoids were positively related to the number of fledglings the parents raised (mean = 0.01 [95% credible interval = −0.00; 0.02]; posterior probability >97%; Figure 3c; Table S3). We did not find a statistical effect of baseline corticosterone on fledgling number when considering its interaction with body condition, nor when accounting for the interaction of baseline glucocorticoids and all physiological parameters (i.e., GPX, OXY, ROMs, and body condition) on the mass of fledglings (Figure 3; Tables S3 and S4).

### 3.2 Do glucocorticoids influence reproductive success depending on environmental conditions?

**FIGURE 4:**
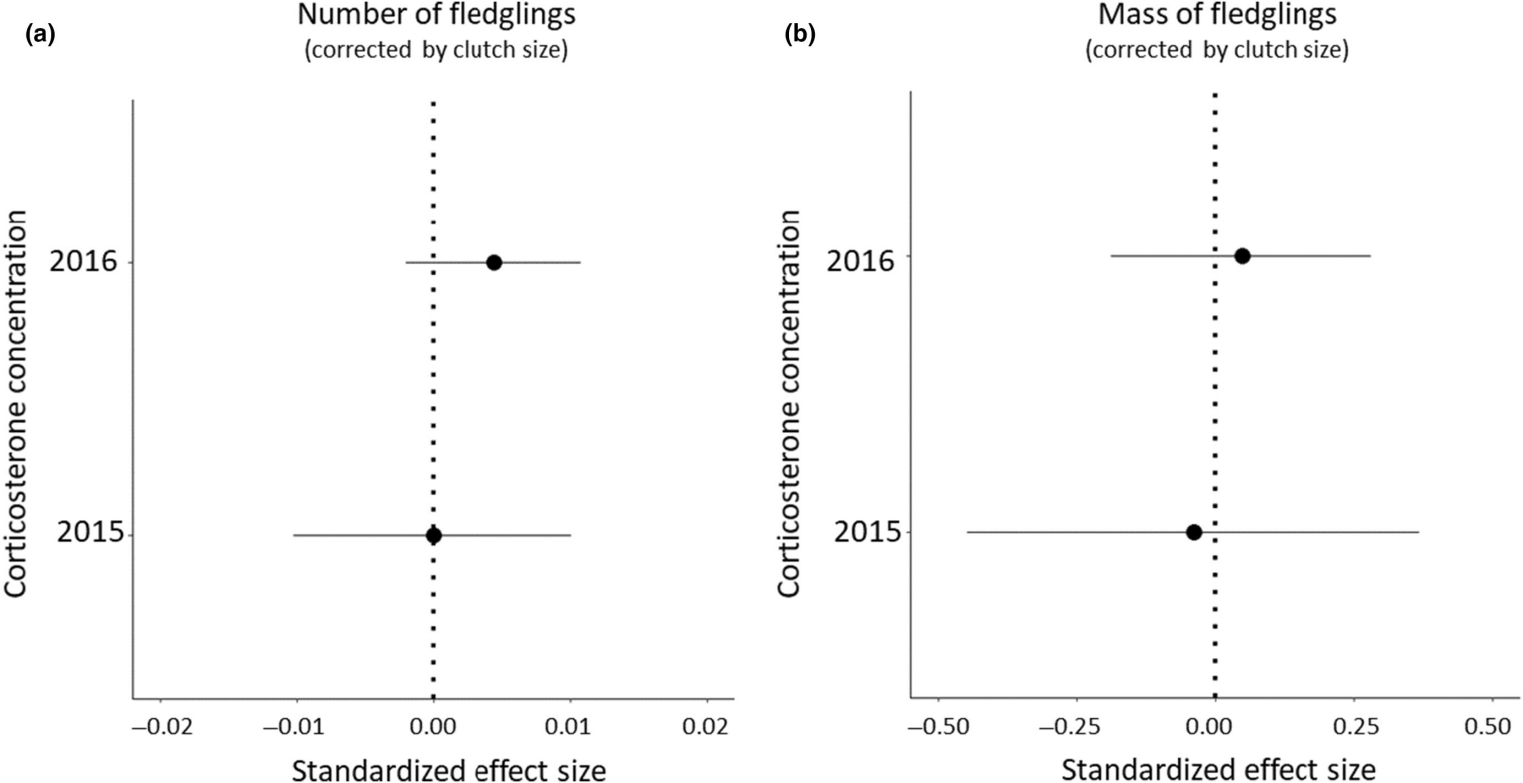
Standardized effect size estimates for the relationship between baseline corticosterone concentration (ng/mL) on the (a) number and (b) mass of fledglings (g) during two breeding seasons. The nestling phase of 2015 was less rainy compared to 2016. Means and lines indicate 95% credible intervals. We found no support for an effect of baseline corticosterone on fledgling number or mass.

The nestling period of 2015 was on average less rainy (and slightly warmer) than the one in 2016 (see above). Despite this, baseline corticosterone concentrations were similar in the 2 years and were not related to the number or mass of the fledglings in either of the 2 years (Figure 4; Tables S5 and S6).

## 4 DISCUSSION

Given the complexity of physiological systems, testing if glucocorticoids are interacting with oxidative status markers and body condition, and if the interaction between glucocorticoids and these other components determines reproductive performance, is not simple. We therefore applied a stepwise analysis by first studying the correlations among the three systems. We found that glucocorticoids are not correlated to oxidative status markers and body condition, similar to the results reported for European starlings (Fowler et al., 2018) and superb starlings (Guindre‐Parker & Rubenstein, 2018). In contrast to these studies conducted under natural conditions, experimentally increased circulating glucocorticoid concentrations were associated with increased oxidative stress and decreased body condition in several, but not all, avian species (e.g., Costantini et al., 2008; Lendvai et al., 2014; Lin et al., 2004a, 2004b; Vágási, Vincze, et al., 2018; but see Majer et al., 2019; Vitousek, Taff, Ardia, et al., 2018). This discrepancy between correlational and experimental studies could be due to experimental manipulations creating more extreme phenotypes than in natural settings (“phenotypic engineering”, Feder et al., 2000; Ketterson et al., 1996), potentially causing an imbalance in other traits and leading to “artificial” physiological interactions. Second, we examined the possibility that even if baseline glucocorticoids were not directly correlated with other physiological traits, they might still be part of an interacting physiological system that together mediates an individual's reproductive fitness. However, we found no relationship between baseline glucocorticoids and reproductive success after accounting for other physiological traits, oxidative status, and body condition. Apart from the current study, three other studies have tested this question, also finding no support for this hypothesis (Fowler et al., 2018; Guindre‐Parker & Rubenstein, 2018; Ouyang et al., 2016). Interestingly, the fitness proxies considered in these four studies include current and future reproduction, as well as adult survival. This suggests that the lack of a relationship that we report here may not be limited to current reproductive success. Third, we tested if the interaction of glucocorticoids with the other two physiological systems influenced reproductive fitness. We found (weak) support for this hypothesis, as baseline glucocorticoids were positively related to fledgling numbers when accounting for their interaction with oxidative status parameters. We also observed that the strength of this relationship was more pronounced for those individuals who also had high concentrations of the enzymatic antioxidant GPX.

Glucocorticoids facilitate the utilization of energy during times of high metabolic needs, like reproduction, by promoting catabolism of lipids and proteins (e.g., McEwen & Wingfield, 2003; Romero et al., 2009). However, maintaining high concentrations of these hormones over a long period of time can be associated with increased oxidative stress (Haussmann & Marchetto, 2010). In this respect, GPX is a very important enzymatic antioxidant that removes inorganic and organic hydroperoxides produced by cells (Halliwell & Gutteridge, 2007), thus potentially reducing oxidative damage. Moreover, GPX concentrations increase with behavioral activities that are associated with increased metabolic rate. For example, the concentration of GPX was positively associated with nest provisioning in male great tits that experienced increased costs of flight because some wing feathers were experimentally shortened (Casagrande & Hau, 2018). Our finding that adult great tits with increased concentrations of both baseline corticosterone and GPX raised more chicks could indicate that some individuals invested more in parental care by elevating their metabolic rate while at the same time protecting themselves from the resulting immediate oxidative damages. If this were the case, it would be worth studying what happens to those same individuals during subsequent reproductive events (in the same year or the year after), because upregulating baseline glucocorticoids and antioxidant defenses could lead to delayed costs. For instance, those feather‐clipped males that invested more in parental provisioning and upregulated GPX more strongly had a lower survival rate from one breeding season to the next (Casagrande & Hau, 2018). Nevertheless, the small effect size and the correlational nature of our study call for caution as well as experimental confirmation.

Contrary to our second prediction, we did not find a relationship between glucocorticoid levels and reproductive success when studying the birds for two breeding seasons with differing environmental conditions. This result may be surprising since physiological traits are expected to be more likely linked with fitness proxies during challenging times (e.g., Bonier, Martin, et al., 2009), and such relationship was reported in a closely related species (*C. caeruleus*; Henderson et al., 2017). However, our findings align with recent research on female European starlings, which also found no relationship between corticosterone and reproductive performance over 2 years with varying environmental conditions (Fowler et al., 2018). It could be argued that the environmental conditions in our study were not sufficiently challenging for the great tits. Nevertheless, parents in 2016 (i.e., the year with more rain) exhibited signs of coping with high metabolic demands, as indicated by lower levels of nonenzymatic antioxidants (OXY), higher levels of enzymatic antioxidants (GPX), and a trend for poorer body condition compared to parents in 2015 (Table 1). This suggests that great tit parents did experience more challenging conditions in 2016 compared to 2015, but that these conditions may still have remained within a normal range, allowing the birds to cope effectively through evolved mechanisms and physiological adjustments, thereby maintaining their reproductive performance across years. Another possibility is that corticosterone does not directly influence reproductive success, which aligns with the hypothesis that physiological traits do not directly impact fitness traits but instead modulate the effect of behavior on fitness (“physiology‐performance‐behavior‐fitness paradigm”; Careau & Garland, 2012). For instance, as discussed earlier, corticosterone might influence nestling provisioning, thereby affecting fledgling survival. Finally, our study sampled individuals only once, examining the relationship between glucocorticoids and fitness proxies at the population level. While this is a common methodology, it is essential to note that populations and individuals can differ in the correlation structures of traits (Malkoc et al., 2022; Niemelä & Dingemanse, 2018; Taff et al., 2024). Therefore, our study does not provide information on the relationship between individual glucocorticoid concentrations (where natural selection operates) and reproductive success. Indeed, Hau et al. (2022) followed a free‐living population of great tits for 5 years and reported individual differences in endocrine responses to changes in environmental temperature. Thus, if true relationships exist between glucocorticoids and fitness proxies, they may become obscured when examining this question at the population level.

## 5 CONCLUSIONS

Glucocorticoids are thought to be key regulators of reproductive performance, but the evidence backing this concept is inconclusive. Here, we examined this relationship within the framework of two hypotheses suggested to explain this discrepancy. The first proposes that glucocorticoids do not act independently, but act together with other physiological traits, while the second suggests their influence becomes noticeable mainly during tough environmental conditions. Our study on wild great tits provided weak support for the first hypothesis and none for the second. Based on our results, researchers might consider manipulating GPX (enzymatic antioxidant) concentrations to investigate how these affect the relationship between baseline glucocorticoids and reproductive performance. Alternatively, for researchers who prefer correlational studies, we recommend repeatedly sampling the same individuals and assessing the relationship between individual glucocorticoid concentrations and reproductive success.

## AUTHOR CONTRIBUTIONS

**Lucia Mentesana:** Conceptualization (equal); data curation (equal); formal analysis (equal); investigation (equal); methodology (equal); project administration (equal); resources (equal); writing – original draft (equal); writing – review and editing (equal). **Stefania Casagrande:** Methodology (equal); writing – review and editing (equal). **Michaela Hau:** Conceptualization (equal); funding acquisition (equal); resources (equal); supervision (equal); writing – review and editing (equal).

## CONFLICT OF INTEREST STATEMENT

The authors declare that they have no competing interests.

## Supporting information


Tables S1–S6

## Data Availability

All data and code are available in an open access repository, Dryad: https://doi.org/10.5061/dryad.69p8cz997.
