# Supplementary material for: Baseline glucocorticoids alone do not predict reproductive success across years, but in interaction with enzymatic antioxidants
Source: Ecol Evol. 2024 Apr 1;14(4):e11193. doi: 10.1002/ece3.11193 (PMC10985372; doi:10.1002/ece3.11193)
Supplement: Supplementary file 1 — Tables S1–S6 [file ECE3-14-e11193-s001.pdf]

Supporting Information

Table 1: Results from a two linear mixed-effect models estimating variation in the number and mass of fledglings in relation during the two breeding seasons. We used the residuals of a linear regression between the number/mass of the nestlings and clutch size as the dependent variable. We fitted 'year' (2015 *vs* 2016) as a fixed factor and 'nest ID' as a random factor. We considered an effect size to be "statistically significant" when the estimated CrI did not overlap the zero.

	Fledgling number (corrected for clutch size)	Fledgling mass (corrected for clutch size)
Fixed factors β (95% CrI)		
Intercept	0.47 (0.29; 0.64)	0.41 (-0.48; 1.31)
Year	-0.07 (-0.26; 0.13)	-0.46 (-1.49; 0.58)
Random factor σ2 (95% CrI)		
Nest ID	0.09 (0.09; 0.10)	2.06 (1.72; 2.48)
Residual variance	0.00 (0.00; 0.00)	1.39 (1.19; 1.62)

Table 2: Results from two linear mixed-effect models testing if glucocorticoids predict reproductive success while accounting for the influence of oxidative status markers and body condition. In each model, we fitted fledgling number and mass corrected by clutch size (i.e., using the residuals of a linear regression of fledgling number or mass and clutch size on day 15 as the dependent variable) as response variables. We scaled and included all five physiological variables as covariates and 'nest ID' as random factor. We considered an effect size to be "statistically significant" when the estimated CrI did not overlap the zero. Oxidative status markers: OXY, non-enzymatic antioxidants in plasma; GPX, enzymatic antioxidant in red blood cells; ROMs, reactive oxygen metabolites in plasma.

	Fledgling number (corrected for clutch size)	Fledgling mass (corrected for clutch size)
Fixed factors β (95% CrI)		
Intercept	0.42 (0.34; 0.49)	0.07 (-0.38; 0.52)
Baseline corticosterone	0.01 (-0.00; 0.02)	0.05 (-0.16; 0.25)
OXY	0.01 (-0.00; 0.02)	0.06 (-0.23; 0.34)
GPX	0.00 (-0.00; 0.01)	0.03 (-0.16; 0.21)
ROMs	0.00 (-0.01; 0.01)	-0.09 (-0.32; 0.12)
Body condition	0.00 (-0.00; 0.01)	0.05 (-0.15; 0.25)
Random factor σ2 (95% CrI)		
Nest ID	0.09 (0.09; 0.09)	1.95 (0.59; 2.38)
Residual variance	0.00 (0.00; 0.00)	0.41 (1.22; 1.66)

Table 3: Results from two linear mixed-effect models testing if glucocorticoids interact with oxidative status markers to jointly predict reproductive success. In each model, we fitted fledgling number and mass corrected by clutch size (i.e., using the residuals of a linear regression of fledgling number or mass and clutch size on day 15 as the dependent variable) as response variables. We included the interaction between baseline corticosterone and the three parameters of oxidative status as covariates and 'nest ID' as random factor. We scaled all covariates. We considered an effect size to be "statistically significant" when the estimated CrI did not overlap the zero. Oxidative status markers: OXY, non-enzymatic antioxidants in plasma; GPX, enzymatic antioxidant in red blood cells; ROMs, reactive oxygen metabolites in plasma.

	Fledgling number (corrected for clutch size)	Fledgling mass (corrected for clutch size)
Fixed factors β (95% CrI)		
Intercept	0.38 (0.34; 0.49)	0.06 (-0.39; 0.51)
Baseline corticosterone	**0.01 (0.00; 0.02)**	0.06 (-0.16; 0.28)
OXY	0.01 (-0.00; 0.02)	0.05 (-0.23; 0.33)
GPX	0.01 (-0.00; 0.01)	0.03 (-0.16; 0.22)
ROMs	0.00 (-0.01; 0.01)	-0.12 (-0.35; 0.11)
Baseline corticosterone * OXY	0.00 (-0.01; 0.01)	0.09 (-0.18; 0.36)
Baseline corticosterone * GPX	0.01 (-0.00; 0.02)	-0.05 (-0.23; 0.14)
Baseline corticosterone * ROMs	-0.00 (-0.00; 0.01)	-0.01 (-0.22; 0.19)
Random factor σ2 (95% CrI)		
Nest ID	0.09 (0.09; 0.09)	1.94 (1.57; 2.37)
Residual variance	0.00 (0.00; 0.00)	1.42 (1.22; 1.66)

Table 4: Results from two linear mixed-effect models testing if glucocorticoids interact with body condition to jointly predict reproductive success. In each model, we fitted fledgling number and mass corrected by clutch size (i.e., using the residuals of a linear regression of fledgling number or mass and clutch size on day 15 as the dependent variable) as response variables. We included the interaction between baseline corticosterone and body condition as covariates and 'nest ID' as random factor. We scaled all covariates. We considered an effect size to be "statistically significant" when the estimated CrI did not overlap the zero.

	Fledgling number (corrected for clutch size)	Fledgling mass (corrected for clutch size)
Fixed factors β (95% CrI)		
Intercept	0.42 (0.34; 0.49)	0.07 (-0.39; 0.53)
Baseline corticosterone	0.01 (-0.02; 0.02)	0.03 (-0.17; 0.23)
Body condition	0.00 (-0.00; 0.01)	0.04 (-0.16; 0.24)
Baseline corticosterone * Body condition	-0.00 (-0.01; 0.01)	0.01 (-0.19; 0.21)
Random factor σ2 (95% CrI)		
Nest ID	0.09 (0.09; 0.09)	2.05 (1.70; 2.43)
Residual variance	0.00 (0.00; 0.00)	1.40 (1.20; 1.64)

Table 5: Results from two linear mixed-effect models testing if that baseline corticosterone influences reproductive fitness traits in a context-dependent way. In each model, we fitted fledgling number and mass corrected by clutch size (i.e., using the residuals of a linear regression of fledgling number or mass and clutch size on day 15 as the dependent variable) as response variables. We included baseline corticosterone as a covariate, 'year' (2015 *vs* 2016) as a fixed factor, along with their interaction, and 'nest ID' as random factor. We considered an effect size to be "statistically significant" when the estimated CrI did not overlap the zero.

	Fledgling number (corrected for clutch size)	Fledgling mass (corrected for clutch size)
Fixed factors β (95% CrI)		
Intercept	0.47 (0.29; 0.64)	0.43 (-0.49; 1.31)
Baseline corticosterone	0.00 (-0.02; 0.02)	-0.04 (-0.46; 0.36)
Year	-0.07 (-0.27; 0.12)	-0.47 (-1.49; 0.59)
Baseline corticosterone * Year	0.00 (-0.01; 0.03)	0.09 (-0.37; 0.56)
Random factor σ2 (95% CrI)		
Nest ID	0.09 (0.09; 0.10)	2.06 (1.70; 2.48)
Residual variance	0.00 (0.00; 0.00)	1.39 (1.20; 1.63)

Table 6: Results from linear models testing the differences in each of the five physiological parameters between the two years. In each model, we fitted ‘year’ (2015 *vs* 2016) as a fixed factor. We considered an effect size to be “statistically significant” when the estimated CrI did not overlap the zero. Oxidative status markers: OXY, non-enzymatic antioxidants in plasma; GPX, enzymatic antioxidant in red blood cells; ROMs, reactive oxygen metabolites in plasma.

	Baseline corticosterone	OXY	GPX	ROMs	Body condition
Fixed factors β (95% CrI)					
Intercept	5.17 (4.02; 6.31)	0.51 (0.09; 0.92)	-0.48 (-0.89; -0.07)	-0.08 (-0.51; 0.35)	0.30 (-0.84; 0.08)
Year	-0.19 (-1.44; 1.11)	**-0.64 (-1.10; -0.17)**	**0.60 (0.14; 1.05)**	0.10 (-0.38; 0.57)	-0.38 (-0.84; 0.08)
